# Assessing the efficiency of methods to teach adolescents about testicular cancer and testicular self-examination

**DOI:** 10.55730/1300-0144.6101

**Published:** 2025-10-26

**Authors:** Ayşe Gül GÜVEN, Laden JAFARİ, Murat Doğuş GÜNEL, Özlem AKBULUT

**Affiliations:** 1 Division of Adolescent Medicine, Department of Pediatrics, Faculty of Medicine, Ankara University, Ankara, Turkiye; 2 Division of Adolescent Medicine, Department of Pediatrics, Bayındır Hospital, Ankara, Turkiye; 3 Department of Biostatistics, Faculty of Medicine, Ankara University, Ankara, Turkıye; 4 Division of Adolescent Medicine, Department of Pediatrics, Faculty of Medicine, Başkent University, Ankara, Turkiye

**Keywords:** Adolescents, testicular cancer, self-examination, male

## Abstract

**Background/aim:**

Due to the increasing incidence of testicular cancer (TC), early testicular self-examination (TSE) in adolescent males is crucial for early detection and treatment. However, it is uncertain which method is best for teaching about TC and TSE in this age group. This study aimed to assess the effectiveness of formal (face to face with model) and informal (video presentation) approaches in male adolescents about TC and TSE.

**Material and methods:**

This intervention study was conducted in 2 high schools, and included a study group (n = 142) and a control group (n = 102). In the study group, a video was shown to one subgroup of male students (n = 60) about testicular anatomy, cancer, and self-examination, while another subgroup (n = 82) received in-person training on the same topics using a model.

**Results:**

The effectiveness of both trainings was evaluated at the end of the third month. Both trainings were significantly effective (p < 0.05). The number of male adolescents performing TSE increased significantly in both training groups (p < 0.001).

**Conclusion:**

Both video content tailored to the developmental level of the male adolescent group and face-to-face training using interactive and professionally produced medical models were effective in teaching about TC and TSE and can lead to beneficial behavioral changes in performing TSE.

## 1. Introduction

Adolescence is a critical period during which individuals develop body awareness, risk-taking tendencies, and preventive health behaviors that can shape their future well-being in adulthood. Neglecting adolescent health not only threatens previous gains in child health but can also negatively affect adult health, exacerbate health inequalities, and contribute to broader social disparities [1]. Adolescent boys and young men, in particular, have the highest rates of suicide and homicide, as well as unintentional injuries. They also show some of the highest rates of substance abuse and receive up to 80% less basic preventive care, including regular check-ups and health insurance, compared to girls [2]. Societal ideals of masculinity, along with pressures on adolescent boys to appear confident, tough, and resilient, have been linked to poor sexual health outcomes, reduced utilization of health services, and increased rates of preventable diseases such as testicular cancer (TC) [2].

TC is the most common solid tumor in adolescent males and is the most common cancer in this age group, with 73% of patients surviving 5 years if detected and treated early^1^. According to data from the Turkish Ministry of Health, the cancer incidence rates in males aged 10–14 years and 15–19 years were 0.4 and 4.1 per 100,000, respectively, between 2016 and 2020^2^. Adolescents continue to have worse survival rates than children and adults [3]. To address this, the American Academy of Pediatrics advises adding a testicular examination to detect cancer in patient interviews for health maintenance beginning at age 11^3^. However, national pediatric, oncological, and public health organizations have varied approaches to using testicular self-examination (TSE) to screen for TC^4^,^5^. The Society for Adolescent Health and Medicine [4] recommends that males have regular testicular examinations to detect TC and other anomalies. Education about TSE may facilitate the early detection of TC and enhance young men’s understanding of their bodies, thereby encouraging them to take responsibility for their health [5]. Building on this, it is important to determine which educational methods are most effective in improving adolescents’ knowledge, awareness, and practices regarding TSE.

Previous studies have shown that informal educational methods—such as videos, booklets, shower cards, or online materials—can be effective, particularly when learners have limited opportunities to interact with an instructor. Nevertheless, interactive, face-to-face interventions have also lead to strong outcomes and are often associated with positive behavioral changes [6]. The present study aimed to assess male adolescents’ awareness and knowledge of TC and TSE, evaluate their practices, and identify the most effective modern method for educating them about TC and encouraging regular TSE. This is particularly important given the rising incidence of TC in recent years [7]. Accordingly, we sought to compare the effectiveness of video-based instruction and face-to-face model-based training, hypothesizing that the latter would yield better results.

## 2. Methods

### 2.1. Participants

This intervention study was conducted between January and May 2023 in 2 public high schools in Ankara, involving 10th-grade male students. Written informed consent was obtained from both the adolescents and their parents after they were informed about the study by teachers and the researchers. Ethical approval was obtained from the Ethics Committee of Ankara Training and Research Hospital (decision number: 1057).

### 2.2. Intervention

Participants from 2 schools were randomly assigned to 3 groups: video/model training (study) and control (no training). The video group (n = 60) were taught using a video created by the authors. The content was developed by reviewing literature on testicular anatomy (e.g., penis, scrotum, testis, epididymis, vas deferens, spermatic cord), TC, and TSE techniques. Care was taken to ensure that the video content provided clear explanations that were specific to adolescents and was scheduled to last 10 min. Participants were asked to complete study forms immediately before and 3 months after the training.

The authors delivered similar instructional content to the second group (n = 82) in clusters of 10 participants, using a direct teaching method through a face-to-face interactive approach. This training incorporated the use of a testicular examination model, similar to those utilized by urologists in outpatient clinical settings. Guidance counselors escorted participants to the training room one at a time from their courses to avoid them influencing each other on the training topic. Similar content in the video presentation was explained understandably by a single narrator author using a model and expressions appropriate for the adolescents’ developmental level. It was stated that students could ask any questions they had during or after the presentation. The training time with the model averaged 10 min. Participants were asked to complete study forms before and 3 months after the training.

Participants in the control group (n = 102) were asked to complete the study questionnaires and were randomly selected from males at a similar grade level to the study group in both schools. The authors and teachers were present as observers throughout the entire process of form completion in the schools, ensuring that participants did not influence one another while filling out the forms.

### 2.3. Measures

After ensuring confidentiality, the adolescent was asked to complete the following forms.

#### 2.3.1. Form 1: Demographic information form

At baseline, students in the study (video/model) and control groups completed a general information form developed by the authors, covering demographics, family history, prior TSE knowledge, and whether they had consulted a doctor for testicular issues.

#### 2.3.2. Form 2: Testicular self-examination practice assessment form

At baseline, all groups completed a TSE evaluation form developed from literature. This form included the following questions:

1. Have you heard of testicular self-examination?
2. Have you ever performed a testicular self-examination before?
3. If so, how frequently did you do it? a) Once per month, b) once every 3 months, c) once every 6 months, or d) once a year or less frequently.
4. Have you ever had your testicles examined by a doctor?
5. Instead of self-examination, I prefer to be examined by a health professional regularly.
6. Do you feel anxious when performing a testicular self-examination?
7. Except for question 3, all answers were yes/no.

#### 2.3.3. Form A: Testicular cancer knowledge and awareness assessment form

At baseline, the video/model and control groups completed a 5-point Likert scale form assessing knowledge and awareness of TC. The video/model group repeated this at month three. This form included the following questions:

1. The most common age group for testicular cancer is adolescents and young adults (15–45 years old).
2. Early detection increases the likelihood of successful treatment for testicular cancer.
3. Detecting testicular cancer at an early stage raises treatment rates by more than 90%.
4. People with a family history of testicular cancer are more likely to develop the disease.
5. There could be considerable variation between testicles.
6. A testicular mass is a strong indicator of testicular cancer.
7. Testicular cancer is curable.
8. Testicular cancer undoubtedly causes infertility.
9. People my age are not likely to have testicular cancer.
10. Testicular cancer patients’ sexual lives come to an end.

Responses were organized as follows: Don’t know (DN), strongly disagree (SD), disagree (D), agree (A), strongly agree (SA).

#### 2.3.4. Form B: Testicular self-examination knowledge and awareness assessment form

At baseline, all groups completed a 5-point Likert scale form assessing TSE knowledge and awareness. The video/model group repeated the form at month three (see Form B in appendix). The contents of the form were as follows:

1. A self-testicular examination should be done at regular intervals.
2. Self-examination allows people to discover testicular cancer at an early stage.
3. During the testicle examination, it is determined whether the testicles are in place, if they are painful, if there is a color change, if there is a noticeable variation in size, and if any further swelling, bulk, or roughness can be felt on the testicles.
4. Regular testicular examination is critical for early identification of testicular disorders.
5. A self-testicular examination should be done after showering.
6. I avoid doing testicular self-exams because I am terrified of discovering something.
7. The self-testicular examination is a painful practice.

Responses were organized as follows: Don’t know (DN), strongly disagree (SD), disagree (D), agree (A), strongly agree (SA).

### 2.4. Statistical analyses

Data were analyzed using IBM SPSS Statistics version 22. A 2-sided Wilcoxon signed-rank test (α = 0.05) was applied to assess paired differences before and after training. Sample size per group was determined as 57 (effect size = 0.5, power = 95%). Chi-square tests examined associations between categorical variables across groups (video, model, and control). The Kruskal–Wallis test showed a significant age difference between groups (p < 0.001), followed by pairwise comparisons. The McNemar test was used to analyze pre- and posttraining yes/no responses (Form 2, Q1–Q5) in the video and model groups. Responses in Forms A and B were coded to reflect knowledge change, with some items reverse-coded. Pretraining differences among the three groups were tested using the Kruskal–Wallis test. The Wilcoxon test compared pre- and postscores within the video and model groups, while the Mann–Whitney U test compared responses between these two groups.

## 3. Results

No significant relationship was found between the three groups (video, model, andcontrol) regarding family structure, parents’ employment and education, family history of testicular disease or cancer, prior TSE information, or doctor consultations for testicular issues (p > 0.05) (Table 1).

At baseline, prior knowledge of testicular examination was significantly related to TSE practice (p < 0.001) across all groups (video, model, and control). Among those who had previously received information about testicular examination, 21.1% performed a TSE, compared to only 1% of those who had not received training. There was also a significant association between previous consultation for testicular problems and TSE (p = 0.005). Among those who had previously consulted a doctor for testicular problems, 30% had performed a TSE, compared with 2.8% of those who had not consulted a doctor. No association was found between the practice of TSE and family history of testicular disease (p = 0.633), paternal education level (p = 0.195), maternal education level (p = 0.195) or family structure (p = 0.394). However, there was an association between the practice of TSE and family history of TC (p = 0.003).

Both video and model trainings resulted in statistically significant increases in “yes” responses regarding TSE practice (p < 0.05). Adolescents’ awareness and practice of TSE improved after both trainings. While both trainings reduced TSE-related stress, further analysis was limited due to insufficient sample size for between-group comparisons (Table 2).

There was no significant difference between the video and model groups in scores on Form A and B before training (p > 0.05). At the end of the training, there was a significant increase in all except 3 questions (A4, A9, and B6) in the video group and in all except 4 questions (A4, A8, A10, and B6) in the model group, i.e. both trainings were found to be significantly effective in educating the participants (p < 0.05). In addition, when comparing the effectiveness of the two trainings, a significant (p < 0.05) increase was observed in the scores for all questions except question 7 in form A in both groups, i.e. no difference was observed between the effectiveness of the two trainings (Table 3).

## 4. Discussion

The primary finding of this study was a significant increase in TSE frequency in both the video-based and model-based training groups. Both groups also showed significant improvements in awareness of TC and self-examination, with no statistically significant difference between the two training methods.

Studies encouraging men to perform TSE are relatively scarce [8–10]. In a recent study conducted among college students pursuing a bachelor’s degree, only 11.4% reported practicing TSE, while 43.3% showed a good level of knowledge on TSE [10]. The baseline rates of practicing TSE in our study were even lower (10% and 5.1% in the video/model groups, respectively), and the proportion of students who had heard about TSE was 30% and 20.3%. Similarly, an Ethiopian study among university students found that 32.4% had heard of TSE and 21.5% had practiced it [11]. These discrepancies among studies may be explained by differences in sociodemographic characteristics, educational level, health literacy, cultural context, and methodological variations. However, the consistently low prevalence across diverse populations emphasizes that TSE awareness and practice remain suboptimal worldwide. Another important common finding is the source of knowledge. Both in our study and in the previous literature, students reported gaining information about TSE primarily through mass media rather than formal education in schools or health institutions. This finding underlines a critical gap in structured health education programs targeting adolescents and young adults. Considering the preventable nature of delayed TC diagnosis, there is a strong need for health policymakers and educators to develop evidence-based, adolescent-friendly educational strategies. Integrating TSE education into school curricula, youth health programs, and primary care services, while simultaneously harnessing the wide reach of mass media, may represent the most effective approach. Future studies should further investigate if school-based, healthcare-based, or media-driven educational intervention result in the most sustainable behavioral change among adolescents and young men.

In a study comparing the effectiveness of a TSE demonstration video with and without implementation intentions among 93 male students aged 18–32, Heverin et al. [12] reported that after 4 and 8 weeks, the addition of implementation intentions did not provide any advantage beyond watching a demonstration video alone. They concluded that video itself was already a highly effective intervention for improving TSE practice. Notably, the videos used in their study were retrieved from YouTube. However, previous evaluations have shown that video content on YouTube directed at adolescents often lacks accuracy, comprehensiveness, and quality [13]. This raises concerns about the reliability of using freely available online content for health education in this sensitive age group. In contrast, our study deliberately used video material that was developed in accordance with up-to-date TC guidelines, prepared by specialists in adolescent health, and narrated in a style appropriate for the developmental stage of adolescents. By doing so, we aimed not only to ensure the accuracy of the content but also to increase the likelihood of sustained behavioral impact. This comparison highlights that while video is an effective medium for promoting TSE, the source and quality of the video are critical in determining the effectiveness of the intervention. Future interventions should therefore prioritize the development of evidence-based, age-appropriate educational materials, rather than relying solely on unverified online resources.

In a 2016 review evaluating the most effective techniques for teaching TSE in adolescents and young men [6], it was reported that almost all professional, formal, or informal methods—except for those relying solely on humor or written material—were significantly effective in increasing both awareness and the practice of TSE. Consistent with these findings, our study showed that both structured (model-based) and informal (video-based) training improved TC knowledge, awareness, and TSE practice among adolescents. From a clinical and public health perspective, these results suggest that TSE education programs can be integrated into school-based health curricula and could be implemented by family physicians and school nurses in primary care settings. Furthermore, TSE training may enhance body awareness and reproductive health confidence in adolescent males, supporting broader adolescent health goals. Ultimately, educational materials such as videos and anatomical models could be adapted and incorporated into national cancer prevention and screening strategies.

Audiovisual elements—particularly video—are an essential component of online learning. Beyond offering significant flexibility, video can serve as a highly effective instructional medium. When used appropriately, media engages students, enhances depth and retention of knowledge, stimulates motivation to learn, and helps illustrate the relevance of the subject matter^6^. Previous studies have shown that video-based education can be highly effective when the content is tailored to educational guidelines and the needs of the target audience^7^. In our study, the video training—developed in line with these principles—proved to be as effective as face-to-face instruction involving a model demonstration by a physician. This finding supports the view that well-structured audiovisual materials may substitute for, or complement, traditional instructor-led training in adolescent health education. Future interventions could focus on developing interactive and age-appropriate video modules that promote active participation and long-term behavioral change among adolescents.

The COVID-19 pandemic necessitated the employment of models other than traditional face-to-face, on-site teaching models. It has been proven that online learning can be just as valuable and effective as face-to-face instruction, as long as the class material is well organized and offered via digital platforms that students may access at any time^8^. Furthermore, studies examining different theories of learning have found that online teaching is more likely to attract students’ attention when both the teacher and the topic are displayed on the screen simultaneously, with the instructor pointing to the location of the content while describing it [14]. The use of video-based learning as a medium for distance learning has been shown to have a positive effect, improving cognitive abilities, and learning performance, developing students’ interest and motivation, and providing students with a better understanding of the concepts taught [15]. Since our study was conducted in the aftermath of the pandemic, when students’ enthusiasm for video education remained high and our video preparation technique was designed to capture their attention, video education may have been as effective as face-to-face instruction. Overall, the effectiveness of video education observed in our study indicates that well-prepared audiovisual materials can be a reliable component of learning even beyond periods of crisis such as the pandemic.

In our study, the posttraining intervention was evaluated only at the third month. In previous studies assessing training methods in this field, both video-based and face-to-face education were evaluated at follow-up intervals ranging from 1 to 6 months [10]. The COVID-19 pandemic had lasting effects on adolescents, including school dropouts and online attendance from home [16]. Additionally, school absences and disruptions caused by the earthquake disaster in Türkiye [17] limited the feasibility of conducting long-term follow-up assessments, resulting in the control evaluation being performed only at the third month. Nevertheless, this time frame was sufficient to evaluate the short-term effectiveness of the training and to observe meaningful behavioral outcomes.

In this study, the control group completed only the initial questionnaire, and no follow-up assessment was conducted after 3 months. This decision was based on previous studies involving adolescents and young men, which showed that even without any educational intervention, participants’ knowledge about TC and their frequency of performing TSE significantly increased after completing baseline questionnaires on health beliefs, knowledge, and attitudes [18,19]. Therefore, evaluating the control group again at the 3-month follow-up could have introduced a confounding effect, as simply completing the questionnaires for a second time might have increased participants’ awareness and knowledge about TC and TSE, even without receiving any formal education. This limitation makes it difficult to determine whether the long-term improvements observed were solely due to the educational interventions or influenced by exposure to other information sources over time. However, given the continuing rise in the incidence of TC and the primary aim of comparing the effectiveness of 2 different educational approaches, the 3-month follow-up assessments in the intervention groups provided valuable comparative data. An important contribution of this study is that it shows the feasibility of implementing TSE education among adolescents. Our findings further suggest that TSE education can be successfully integrated into school-based health programs, where primary care professionals—such as school health nurses and family physicians—can play an active and sustainable role.

## Conclusion

The study found that video- and model-based teaching increased high school students’ understanding of TC and the frequency of performing TSE. We believe that video content tailored to the developmental level of the target group, or training utilizing interactive and professionally designed medical models, may lead to sustained positive health behaviors among adolescents. Future long-term studies involving different age groups would provide clearer evidence on adolescents’ TSE practices and further clarify the effectiveness of TSE education.

## Supplementary Information



## Figures and Tables

**Table 1:** Comparison of demographics between video and model training participants and control group.

		Video (n = 60)	Model (n = 82)	Control (n = 102)	p
Family Structure	Core	55 (91.7)	77 (93.9)	91 (90.1)	0.812
	Large	2 (3.3)	2 (2.4)	2 (2.0)	
	Divorced	2 (3.3)	3 (3.7)	7 (6.9)	
	One of the parents died	1 (1.7)	0 (0.0)	1 (1.0)	
Mother’s employment status(working)		38 (64.4)	45 (54.9)	58 (57.4)	0.514
Father’s employment status (working)		56 (93.3)	76 (92.7)	95 (94.1)	0.932
Mother’s grade at school	Illiterate	0 (0.0)	0 (0.0)	0 (0.0)	0.286
	Literate	1 (1.7)	1 (1.2)	0 (0.0)	
	Primary-middle school	4 (6.8)	6 (7.3)	8 (7.9)	
	High School	13 (22)	16 (19.5)	23 (22.8)	
	Graduate	37 (62.7)	50 (61)	49 (48.5)	
	Postgraduate	4 (6.8)	9 (11)	21 (20.8)	
Father’s grade at school	Illiterate	0 (0.0)	0 (0.0)	0 (0.0)	0.139
	Literate	1 (1.7)	0 (0.0)	0 (0.0)	
	Primary-middle school	4 (6.7)	3 (3.7)	6 (5.9)	
	High School	11 (18.3)	9 (11.0)	20 (19.8)	
	Graduate	37 (61.7)	51 (62.2)	48 (47.5)	
	Postgraduate	7 (11.7)	19 (23.2)	27 (26.7)	
Does anyone in the family have a testicular disease?	No	56 (94.9)	76 (95)	82 (86.3)	0.201
	Yes	1 (1.7)	1 (1.3)	6 (6.3)	
	I don’t know	2 (3.4)	3 (3.8)	7 (7.4)	
Is there testicular cancer in the family?	No	58 (98.3)	78 (97.5)	88 (93.6)	0.458
	Yes	0 (0.0)	1 (1.3)	1 (1.1)	
	I don’t konow	1 (1.7)	1 (1.3)	5 (5.3)	
Have you been informed about testicular examination before? (Yes)		14 (23.3)	8 (9.9)	16 (17.2)	0.096
If so, where did you get this information?	School	2 (3.3)	2 (3.4)	0 (0.0)	0.213
	Mass media	7 (11.7)	5 (6.1)	12 (11.8)	0.378
	Health institution	6 (10)	2 (2.4)	4 (3.9)	0.100
Have you ever consulted a doctor due to testicular problems? (No)		57 (96.6)	74 (94.9)	86 (95.6)	0.886

* Frequency (percentage), excluding the age variable, corresponds to mean ± standard deviation [median (minimum – maximum)] values for the age variable.

**Table 2:** Analysis of “yes” answers given by participants who received video and model training to questions about TSE practices before and after training.

		Video						Model					
Questions in Form 2		Before training		After training		p		Before training		After training		p	
1. Have you heard of testicular self-examination? (Yes)		18 (30)		54 (90)		<0.001**		16 (20.3)		78 (98.7)		**<0.001** **	
2. Have you ever performed a testicular self-examination before? (Yes)		6 (10)		22 (36.7)		<0.001**		4 (5.1)		56 (70.9)		**<0.001** **	
3. If so, how frequently did you do it?													
Once per month		1 (1.7)		6 (10)		0.063		0 (0.0)		23 (29.1)		NA		
Once every three months		0 (0.0)		7 (11.7)		NA		2 (2.5)		17 (21.5)		**<0.001** **		
Once every six months		1 (1.7)						0 (0.0)						
Once a year or less frequently		4 (6.7)						2 (2.5)						
4. Have you ever had your testicles examined by a doctor? (Yes)		12 (20)		13 (21.7)		1.000		6 (7.6)		12 (15.2)		0.070		
5.Instead of self-examination, I prefer to be examined by a health professional on a regular basis. (Yes)		43 (71.7)		40 (66.7)		0.741		40 (50.6)		30 (38)		0.121		
6. Do you feel anxious when performing a testicular self-examination?														
Yes		16 (26.7)		9 (15)		NA		17 (21.5)		13 (16.5)		NA		
I don’t know		1 (1.7)		0 (0.0)				4 (5.1)		0 (0.0)				
No		43 (71.6)		51 (85)				58 (73.4)		66 (83.5)				

* p < 0.05;

** p < 0.001

Values for frequency (%) are given for responses that are positive to the questions.

The McNemar test was used to determine the p-value for each question.

**Table 3:** Evaluation of effectiveness before, after, and between trainings for Form A and B questions.

Questions in Form A and B		Video M ± St. Dev. [ Med. (Min–Max) ]	Model M ± St. Dev. [ Med. (Min–Max) ]	p
A1: The most common age group for testicular cancer is adolescents and young adults (15–45 years old).	Before Training	2.3 ± 1.46 [ 1 (1–5) ]	2.0 ± 1.50 [ 1 (1–5) ]	0.219
	After Training	2.98 ± 1.57 [ 4 (1–5) ]	2.91 ± 1.58 [ 4 (1–5) ]	0.859
	p	**0.012**	**<0.001**	
A2: Early detection increases the likelihood of successful treatment for testicular cancer.	Before Training	3.1 ± 1.62 [ 4 (1–5) ]	3.1 ± 1.65 [ 4 (1–5) ]	0.911
	After Training	4.00 ± 1.29 [ 4 (1–5) ]	4.04 ± 1.24 [ 4 (1–5) ]	0.92
	p	**<0.001**	**<0.001**	
A3: Detecting testicular cancer at an early stage raises treatment rates by more than 90%	Before Training	3.1 ± 1.66 [ 4 (1–5) ]	2.8 ± 1.73 [ 4 (1–5) ]	0.288
	After Training	3.82 ± 1.28 [ 4 (1–5) ]	3.59 ± 1.46 [ 4 (1–5) ]	0.528
	p	**0.002**	**<0.001**	
A4: People with a family history of testicular cancer are more likely to develop the disease.	Before Training	2.9 ± 1.60 [ 4 (1–5) ]	3.0 ± 1.56 [ 4 (1–5) ]	0.563
	After Training	3.25 ± 1.53 [ 4 (1–5) ]	3.24 ± 1.49 [ 4 (1–5) ]	0.796
	p	0.121	0.173	
A5: There could be considerable variation between testicles.	Before Training	3.1 ± 1.58 [ 4 (1–5) ]	2.7 ± 1.46 [ 3 (1–5) ]	0.064
	After Training	3.63 ± 1.02 [ 4 (1–5) ]	3.52 ± 1.21 [ 4 (1–5) ]	0.676
	p	**0.03**	**<0.001**	
A6: A testicular mass is a strong indicator of testicular cancer.	Before Training	2.9 ± 1.62 [ 4 (1–5) ]	2.5 ± 1.59 [ 1 (1–5) ]	0.08
	After Training	3.78 ± 1.34 [ 4 (1–5) ]	3.48 ± 1.38 [ 4 (1–5) ]	0.084
	p	**<0.001**	**<0.001**	
A7: Testicular cancer is curable.	Before Training	2.9 ± 1.61 [ 4 (1–5) ]	2.9 ± 1.54 [ 4 (1–5) ]	0.756
	After Training	4.00 ± 1.28 [ 4 (1–5) ]	3.64 ± 1.38 [ 4 (1–5) ]	**0.036**
	p	**<0.001**	**0.001**	
A8: Testicular cancer undoubtedly causes infertility.	Before Training	4.3 ± 0.96 [ 5 (2–5) ]	4.1 ± 1.13 [ 5 (1–5) ]	0.529
	After Training	3.55 ± 1.25 [ 4 (1–5) ]	3.84 ± 0.97 [ 4 (1–5) ]	0.264
	p	**<0.001**	0.068	
A9: People my age are not likely to have testicular cancer.	Before Training	4.0 ± 0.97 [ 4 (1–5) ]	4.2 ± 0.96 [ 4.5 (1–5) ]	0.264
	After Training	3.78 ± 1.06 [ 4 (1–5) ]	3.68 ± 0.77 [ 4 (2–5) ]	0.248
	p	0.217	<0.001	
A10: Testicular cancer patients’ sexual lives come to an end.	Before Training	4.2 ± 1.00 [ 5 (2–5) ]	3.9 ± 1.26 [ 4.5 (1–5) ]	0.231
	After Training	3.57 ± 1.14 [ 4 (1–5) ]	3.79 ± 0.98 [ 4 (1–5) ]	0.359
	p	**<0.001**	0.434	
B1: A self-testicular examination should be done at regular intervals.	Before Training	2.0 ± 1.58 [ 1 (1–5) ]	2.2 ± 1.47 [ 1 (1–5) ]	0.507
	After Training	3.76 ± 1.26 [ 4 (1–5) ]	3.73 ± 1.23 [ 4 (1–5) ]	0.671
	p	**<0.001**	**<0.001**	
B2: Self-examination allows people to discover testicular cancer at an early stage.	Before Training	2.6 ± 1.40 [ 3 (1–5) ]	2.4 ± 1.52 [ 1 (1–5) ]	0.502
	After Training	3.72 ± 1.28 [ 4 (1–5) ]	3.47 ± 1.30 [ 4 (1–5) ]	0.182
	p	<**0.001**	**<0.001**	
B3: During the testicle examination, it is determined whether the testicles are in place, if they are painful, if there is a color change, if there is a noticeable variation in size, and if any further swelling, bulk, or roughness can be felt on the testicles.	Before Training	2.5 ± 1.65 [ 1 (1–5) ]	2.3 ± 1.65 [ 1 (1–5) ]	0.463
	After Training	3.79 ± 1.41 [ 4 (1–5) ]	4.01 ± 1.11 [ 4 (1–5) ]	0.743
	p	**<0.001**	**<0.001**	
B4: Regular testicular examination is critical for early identification of testicular disorders.	Before Training	3.3 ± 1.56 [4 (1–5) ]	3.3 ± 1.52 [ 4 (1–5) ]	0.774
	After Training	3.97 ± 1.03 [ 4 (1–5) ]	4.00 ± 1.04 [ 4 (1–5) ]	0.775
	p	**0.006**	**<0.001**	
B5: A self-testicular examination should be done after showering.	Before Training	1.8 ± 1.42 [ 1 (1–5) ]	1.4 ± 1.04 [ 1 (1–5) ]	0.078
	After Training	3.41 ± 1.45 [ 4 (1–5) ]	3.21 ± 1.53 [ 4 (1–5) ]	0.433
	p	**<0.001**	**<0.001**	
B6: I avoid doing testicular self-exams because I am terrified of discovering something.	Before Training	2.4 ± 1.15 [ 2 (1–5) ]	2.3 ± 1.16 [ 2 (1–5) ]	0.788
	After Training	2.52 ± 1.10 [ 2 (1–5) ]	2.36 ± 0.83 [ 2 (1–5) ]	0.579
	p	0.565	0.441	
B7: The self-testicular examination is a painful practice.	Before Training	4.5 ± 0.91 [ 5 (1–5) ]	4.4 ± 0.98 [ 5 (1–5) ]	0.633
	After Training	3.79 ± 1.18 [ 4 (1–5) ]	3.63 ± 0.97 [ 4 (1–5) ]	0.223
	p	**0.002**	**<0.001**	

Note: Likert scale (1–5): 1 = Don’t know (DN), 2 = Strongly disagree (SD), 3 = Disagree (D) 4 = Agree (A), 5 = Strongly agree (SA).

## Data Availability

The data supporting the findings of this study are available from the corresponding author upon request.

## Footnotes

1
https://www.cancer.org/cancer/types/testicular-cancer/about/key-statistics.html

2
https://hsgm.saglik.gov.tr/depo/birimler/kanser-db/Dokumanlar/Istatistikler/Turkiye_Kanser_Istatistikleri_2020.pdf

3
https://ewscripps.brightspotcdn.com/0c/91/11fa2d9042a19090103467afdf8f/bright-futures-pocket-guide-3rd-edition-1.pdf

4
https://www.uspreventiveservicestaskforce.org/uspstf/document/%20RecommendationStatementFinal/testicular-cancer-screening

5
https://www.cancer.org/cancer/testicularcancer/moreinformation/%20doihavetesticularcancer/do-i-have-testicular-cancer-self-exam.html

6
https://serc.carleton.edu/sp/library/media/index.html

7
https://up.csail.mit.edu/other-pubs/las2014-pguo-engagement.pdf

8
https://peer.asee.org/face-to-face-and-e-learning-styles-for-undergraduate-engineering-technology-students-during-covid-19-pandemic
