# Supplementary material for: Assessing the efficiency of methods to teach adolescents about testicular cancer and testicular self-examination
Source: Turk J Med Sci. 2025 Oct 26;55(6):1435–44. doi: 10.55730/1300-0144.6101 (PMC12779074; doi:10.55730/1300-0144.6101)
Supplement: Supplementary file 1 [file tjmed-55-06-1435-Appendix.docx]

**Form B: Testicular self-examination knowledge and awareness assessment form**

The contents of the form were as follows:

1. A self-testicular examination should be done at regular intervals.

2. Self-examination allows people to discover testicular cancer at an early stage.

3. During the testicle examination, it is determined whether the testicles are in place, if they are painful, if there is a color change, if there is a noticeable variation in size, and if any further swelling, bulk, or roughness can be felt on the testicles.

4. Regular testicular examination is critical for early identification of testicular disorders.

5. A self-testicular examination should be done after showering.

6. I avoid doing testicular self-exams because I am terrified of discovering something.

7. The self-testicular examination is a painful practice.

Responses were organized as follows: Don't know (DN) Strongly disagree (SD) Disagree (D) Agree (A)Strongly agree (SA)
